# How Internet Websites Portray Herbal Vitality Products Containing *Eurycoma longifolia Jack*: An Evaluation of the Quality and Risks of Online Information

**DOI:** 10.3390/ijerph191911853

**Published:** 2022-09-20

**Authors:** Mohd Shahezwan Abd Wahab, Nurfarah Nadiah Abd Hamid, Ali Omar Yassen, Mohd Javed Naim, Javed Ahamad, Nur Wahida Zulkifli, Farhana Fakhira Ismail, Muhammad Harith Zulkifli, Khang Wen Goh, Long Chiau Ming

**Affiliations:** 1 Faculty of Pharmacy, Universiti Teknologi MARA (UiTM) Cawangan Selangor, Kampus Puncak Alam, Puncak Alam 42300, Malaysia; 2 Non-Destructive Biomedical and Pharmaceutical Research Centre, Smart Manufacturing Research Institute, Universiti Teknologi MARA (UiTM) Cawangan Selangor, Kampus Puncak Alam, Puncak Alam 42300, Malaysia; 3 Department of Clinical Pharmacy and Pharmacy Management, Tishk International University, Erbil 44001, Iraq; 4 Department of Pharmaceutical Chemistry, Tishk International University, Erbil 44001, Iraq; 5 Department of Pharmacognosy, Tishk International University, Erbil 44001, Iraq; 6 Faculty of Data Science and Information Technology, INTI International University, Nilai 71800, Malaysia; 7 PAP Rashidah Sa’adatul Bolkiah Institute of Health Sciences, Universiti Brunei Darussalam, Gadong BE1410, Brunei

**Keywords:** Tongkat Ali, herbal medicine, men’s health, health informatic, human and health, public health

## Abstract

Background: Tongkat Ali (TA) or *Eurycoma longifolia* is a herbal medicine (HM) plant traditionally used to treat sexual dysfunction and enhance libido in men. Websites containing information about HM are abundant. However, studies have shown that in general the quality of websites containing information on HM is low. The present study aims to assess the quality and risks of websites containing information about TA supplements and to identify the health claims for TA. Methodology: A cross-sectional study to evaluate the quality and risks of websites discussing TA supplements was conducted. Online marketing websites, research articles, news articles, personal opinions, and those restricted by password were excluded. The quality and risks of websites were assessed using a modified DISCERN tool and a set of risk assessment criteria, respectively. The health claims for TA were identified and analyzed using content analysis. Results: Overall, 321 websites met the inclusion criteria and were further evaluated. The overall rating of the quality of the websites was low, with a mean score ± standard deviation of 1.07 ± 0.51. Most websites lacked information that there may be more than one possible treatment choice and did not discuss areas of uncertainty. However, 67.9% (218/321) of the websites received a risk score of zero. A minority of websites (5/321, 1.6%) discouraged the use of conventional medicines. The most common health claims for TA included in the websites related to the enhancement of testosterone level (121/321, 37.7%), treatment of malaria (112/321, 34.9%), and improvement in libido (108/321, 33.6%). Conclusions: Websites containing information about TA supplements generally have a low-quality rating based on a modified DISCERN tool despite having a low-risk score. Government agencies and healthcare professionals (HCPs) must be more proactive in the critique and dissemination of information relating to HM, and in ensuring the safe use of HM among the public and patients.

## 1. Introduction

Tongkat Ali (TA) or *Eurycoma longifolia* is a herbal medicine (HM) plant that grows in Southeast Asian countries such as Malaysia, Indonesia, and Thailand [1]. Traditionally, TA is used as an aphrodisiac, antipyretic, antimalarial agent, antibiotic, and antidiabetic [2,3,4]. However, TA is most commonly recognized as a complementary and alternative medicine (CAM) to treat sexual dysfunction, and enhance libido in men [4]. The pharmacological effects of TA are attributed to its chemical compounds such as eurycomanone and quassinoid [5,6]. Despite various studies showing the pharmacological activities of TA in animal and in vitro studies [7,8,9], there is still a lack of evidence of many of the plant’s traditional uses [2,10]. Several randomized controlled trials (RCTs) have been conducted to investigate the effects of TA intake on stress markers [11], sleep quality [11], quality of life [12], testosterone level [13], and sexual functions [12,13,14] in men. These RCTs showed that TA supplementation may have clinical effects on men’s health. However, more efficacy trials are warranted to further support current evidence [15].

Due to the publication of reports on the potential benefits of TA, it has received major attention worldwide as a men’s health or enhancement supplement and has been marketed globally as a single ingredient or in combination with other herbs in various pharmaceutical dosage forms (e.g., tablets and capsules). According to a study by Balasubramanian et al., TA is one of the most common ingredients included in erectile dysfunction supplements [16]—the others being ginseng, L-arginine, and horny goat weed. A few surveys among the public in Asian countries showed that many people were using TA for health enhancement whether in crude forms or modern pharmaceutical formulations [17,18,19]. Based on previous studies, TA supplements have been reported to be frequently used as self-medication without recommendations from healthcare professionals (HCPs) [17,18].

Since consumers infrequently consult with HCPs about CAM use, they may obtain information about the products from other sources of information [20]. One of the most common sources for the public to obtain health-related information is the Internet [20,21]. Due to the popularity of HM for health enhancement, websites containing information about the products are abundant. Nevertheless, the literature showed that the majority of websites containing information on CAM including HM were generally low quality [22,23].

A good quality website on HM should contain balanced and unbiased information about HM based on reliable sources [24]. The information should not merely be based on personal opinions or testimonials [25]. Additionally, it should discuss the benefits and risks (e.g., adverse effects, drug–supplement interactions, and contraindications) so that consumers can make informed decisions about their HM use [22]. Moreover, the websites should be “safe” by not containing information that deters consumers from using conventional medicines or following clinicians’ advice, and by being predominantly commercial in nature [25].

At present, there is no study that evaluates the quality and risks of websites containing information about TA supplements. Therefore, the present study aims to: (1) assess the quality of websites containing information about TA supplements using a modified DISCERN tool [26]; (2) evaluate the risks of the websites based on a risk score as described by Schmidt and Ernst [25]; and (3) identify the health claims for TA as stated on the websites. Findings from this study could provide insights into the level of quality and risks of websites containing information about TA, a popular HM among the public. They can demonstrate the need to improve the quality of websites containing information on HM, which can promote the safe use of the products among consumers.

## 2. Methods

This was a cross-sectional study evaluating the quality and risks of websites discussing TA supplements. Websites were included if they discussed any aspects of TA supplements for health use in English or Malay language. We excluded online marketing websites, those that consisted of research articles, news articles, and personal opinions or experiences from social media, blogs, and Internet forums. Websites that were restricted by password were also excluded.

### 2.1. Website Identification and Selection

Data were collected in March 2020. Websites were identified using the Google search engine. The search terms used in the Internet search were divided into two themes: (1) Theme 1: herbal plant of interest—“Tongkat Ali” (term 1) and “*Eurycoma longifolia”* (term 2); and (2) Theme 2: uses of the herbal plant—“supplement” (term 1), “herbal medicine” (term 2), “alternative medicine” (term 3), “complementary medicine” (term 4), and “traditional medicine” (term 5). The search terms in Theme 2 were translated into the Malay language and were also used in the Internet search.

Each search term from “Theme 1” was combined with each search term from both the English and Malay version of “Theme 2” resulting in 20 searches overall. In the searching procedures, cookies and user account information were disabled. Consequently, the first 50 websites resulting from each combination of search terms were recorded for further assessment. The evaluation was limited to the first 50 websites since reports showed that Internet users infrequently browse through further sites when searching the web [27,28].

The Internet search procedure resulted in the identification of 1000 websites for evaluation. A total of 679 websites were excluded from the final analysis because they were online retail websites, restricted with a password, scientific articles, contained no information about TA for health purposes, or were duplicates of other websites. After removing these websites, 321 websites were further evaluated.

### 2.2. Assessment of the Quality of Websites Containing Information about TA Supplements

The appraisal form used to assess the quality of websites containing information about TA supplements was adapted from the DISCERN tool [24]. The DISCERN tool is a validated tool that was developed to assess written medical information using 15 questions that address the reliability and details on treatment, and a 16th question for the overall quality rating of the publication [24,26]. Specifically, questions 1 to 8 of the DISCERN tool address the reliability, dependability, and trustworthiness of information in a publication; whereas questions 9 to 15 address specific details about the information on treatment choices. Question 16 is the overall quality rating of a publication by the reviewer. For each DISCERN item, a rating scale of 1 to 5 is used with a higher rating score indicating more of the criteria were fulfilled. Previous studies have shown that the tool has good inter-rater agreement, and good face and content validity [24,29].

Since the original DISCERN tool concerns the quality of written information on treatment choices for a health problem, several items of the tool may have limited relevance for HM. Therefore, previous studies that evaluated the quality of websites on HM removed and modified several items to meet the context of the research [22,30]. In this study, a modified DISCERN tool was developed and used. The modifications included: (1) removing question 2 (*Does it achieve its aims?*); (2) changing the focus on “publication” to “website”, and “treatment” to “TA” in the questions; (3) dividing question 11 (*Does it describe the risks of each treatment?*) into three separate questions that addressed: (i) drug interactions (*Are drug-supplement interactions mentioned?*); (ii) contraindications (*Are contraindications of TA mentioned?*); and (iii) adverse effects (*Are potential adverse effects of TA mentioned?*); and (4) removing item 12 (*Does it describe what would happen if no treatment is used?*), item 13 (*Does it describe how the treatment choices affect overall quality of life?*) and item 15 (*Does it provide support for shared decision-making?*) of the original DISCERN tool since these items emphasize aspects that are more relevant to conventional treatment.

Overall, the modifications resulted in an appraisal tool that consisted of 14 questions (Table 1). The rating for the modified tool utilized the same method of rating as in the original DISCERN tool. The modified DISCERN tool was piloted on 15 websites containing information on TA. The pilot test showed that the modified tool was practical and able to meet the aims of the study.

### 2.3. Assessment of the Risks of Websites Containing Information about TA Supplements

The risks of websites containing information about TA supplements were assessed using a risk score as described by Schmidt and Ernst [25]. The risks of websites were determined based on the following criteria: (1) “*Does the site discourage the use of conventional medicine?*”; (2) “*Does the site discourage adhering to clinician’s advice?*”; (3) “*Does the site provide opinions and experiences or factual details?*”; and (4) “*Does the site provide commercial details?*” One point was given for each positive answer. Therefore, a “risk score” for each website can range from 0 to 4 with the higher score indicating a greater risk.

### 2.4. Identification of Health Claims for TA in the Websites

For each included website, the health claims for TA were identified, recorded, and analyzed using content analysis. Using the content analysis, the health claims for TA were organized and grouped together under an appropriate category.

The rating for the quality of the included websites using the modified DISCERN tool, determination of the risk score, and content analysis of health claims for TA were conducted by NNAH, checked by MSAW, and later discussed among the research team. Any disagreements concerning the rating or data analysis were resolved through discussion.

### 2.5. Statistical Analysis

For the assessment of the quality of websites containing information about TA supplements using the modified DISCERN tool, the mean and standard deviation (SD) for each item was calculated. The risk score of websites and health claims were presented as frequency and percentage.

## 3. Results

Overall, 321 websites were included in the assessment of the quality and risks of websites containing information about TA supplements. All of the websites were also examined to identify the stated health claims for TA. 

Table 1 reports the rating score for each of the modified DISCERN items. The worst performing item was question 13 (*Is it clear from the website that there may be more than one possible treatment choice?*) with a mean score ± SD of 1.02 ± 0.16. Item 7 (*Does the website refer to areas of uncertainty?;* mean score ± SD = 1.05 ± 0.23) and item 1 (*Are the aims clear?*; mean score ± SD = 1.14 ± 0.60) also recorded a low mean score. Items that recorded the highest score were item 9 (*Does the website describe the benefits of TA?*; mean score ± SD = 2.90 ± 1.26) and item 8 (*Does the website describe how TA works?*; mean score ± SD = 2.79 ± 1.31). However, both scores were moderately high, indicating that the criteria were partially fulfilled. The overall quality rating of websites containing information about TA supplements was low, with a mean score ± SD of 1.07 ± 0.51.

### 3.1. Risks of Websites Containing Information about TA Supplements

**Figure 1:**
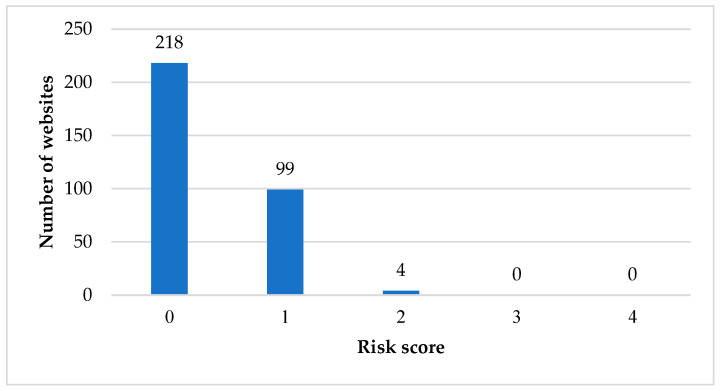
Risk score for the websites evaluated in the study.

Five out of 321 websites (1.6%) directly or indirectly discouraged the use of conventional medicines. Websites that directly discouraged the use of conventional medicines had statements indicating that conventional medicines may cause more harm and side effects than TA supplements. Our data showed that none of the websites discouraged readers from adhering to clinicians’ advice. Two websites (0.6%) included information that was judged as opinions and experiences without factual details. Additionally, 31.5% (101/321) of the websites contained commercial details that promoted the purchase of TA supplements. Overall, 67.9% (218/321) of the websites received a risk score of zero. Approximately, 30.8% (99/321) of the websites had a score of one, whereas four websites (1.2%) had a score of 2. No website received a score of three or above (Figure 1).

### 3.2. Health Claims for TA on the Websites

Table 2 summarizes the health claims for TA supplementation on the websites. The most common health claims described on the websites related to the enhancement of testosterone level (121/321, 37.7%), treatment of malaria (112/321, 34.9%), and improvement in libido (108/321, 33.6%). A small percentage of the websites indicated that TA supplements can enhance energy (59/321, 18.4%), have anticancer properties (57/321, 17.8%), can increase muscle gain (56/321, 17.4%) and treat fever (48/321, 15%).

## 4. Discussion

The present study showed that websites containing information about TA supplements are generally low in quality. These websites generally focus on the benefits of TA supplements but have limited information on the risks of the products, such as drug–supplement interactions, contraindications, and adverse effects.

A low-quality rating based on a modified DISCERN tool was also reported by Thakor et al., for e-commerce websites that sold St. John’s wort (*Hypericum perforatum*) products [22]. In the study, 96% of the websites were reported to have poor quality. Similar to our findings, the majority of e-commerce websites on St. John’s wort did not contain information on drug–supplement interactions, contraindications, or adverse effects [22]. In another study, Baudischova et al., evaluated 199 web domains and 850 websites for the top 100 best-selling dietary supplements (DS) in the Czech Republic using evaluation criteria according to regulatory and clinical points of views [23]. The authors concluded that the quality of information related to DS (including HM) that is available on the Internet in the Czech Republic is generally low [23]. They also reported that only a minority of the websites contained information on the risks of DS (e.g., adverse reactions, drug–supplement interactions, contraindications, or use during pregnancy and breastfeeding).

Previous studies demonstrated that the quality of online and written information on pharmaceutical drugs such as antidepressants and antidiabetics assessed using the DISCERN tool was generally good [31,32]. Thus, the findings reported in the present study highlighted the gap in the quality of HM information on the Internet. This should be a cause for concern since many HM consumers use the Internet to search for information about the products [33,34,35]. As reported in previous studies, many HM consumers have chronic diseases and use conventional medicines [36,37]. Drug–supplement interactions may cause the conventional therapy to fail or adverse reactions to occur [23]. In the context of TA supplements, contraindications for the products have been suggested in children, pregnant/lactating women, men with breast cancer or prostate cancer, patients with diabetes mellitus, heart disease, kidney disease, liver disease, or sleep apnoea, and those with a known allergy or hypersensitivity to TA [4]. Additionally, although there are limited data on the adverse effects associated with TA supplementation, side effects such as insomnia, anxiety, and restlessness have been reported [4]. Moreover, interactions between TA and drugs such as propranolol, antibiotics, and antidiabetics are possible [38,39,40].

Our findings also showed that many websites on TA supplements had limited information about the fact that there may be more than one possible treatment choice, and the majority did not refer to areas of uncertainty. This means that many of the websites did not include a description of who is most likely to benefit from TA supplements, or suggestions for alternatives. Ideally, websites should include information that for certain health conditions such as low testosterone level and erectile dysfunction, a thorough assessment by doctors to investigate the severity and cause of the health conditions should be performed. Treatment for these conditions such as testosterone replacement therapy (for low testosterone) and phosphodiesterase inhibitors such as sildenafil and tadalafil (for erectile dysfunction) should be described [41]. Additionally, the websites should discuss the gaps in knowledge concerning TA supplementation such as limited evidence for efficacy.

Our findings highlight the need for HCPs to become aware of the fact that TA supplement users may access information about TA from websites that might contain inadequate and unbalanced information about the supplements. The websites that are low in quality may not be suitable sources of information for consumers to make informed decisions about TA or other HM use. Thus, it is crucial for HCPs to be more proactive in communicating with patients about their HM use. HCPs should assess the appropriateness of HM use by consumers by identifying drug–supplement interactions, contraindications, and adverse effects, assist them in navigating HM information, and refer them to high-quality resources [42]. Additionally, HCPs may provide consumers with balanced information regarding both the benefits and possible risks of taking HM. This would ensure that HM is being used appropriately and safely [43].

Despite the overall low-quality rating of the websites included in this study, the assessment of the risks of the websites based on a set of risk assessment criteria showed that the majority of the websites had a low-risk score. Additionally, the number of websites containing information that discouraged the use of conventional medicines was low. Only a small minority of the websites stated that conventional medicines are more harmful and have more side effects compared to TA. It should be noted that in this study the risks of websites were evaluated using a scoring method described by Schmidt and Ernst in a study that assessed the risks of websites on CAM for cancer [25], a medical condition that differs from the conditions targeted by TA in terms of disease severity. Despite this, the items contained in the criteria are not specific to cancer. Thus, it can be argued that the risk assessment tool can be applied to websites on CAM for other diseases. Of note, previous studies have utilized the risk assessment tool to assess the risks of websites on CAM for dry eye disease and glaucoma [44,45].

A main concern relating to the risks of the websites was the fact that about one third of them contain commercial details that promoted the purchase of TA supplements. As noted in a recent study conducted by Ng et al., websites on DS for weight loss that were largely commercial in nature were poor quality. Additionally, those websites frequently lacked information about the risks and benefits of the products, and generally provided more biased information [46]. That being said, the present study could not confirm whether the websites containing commercial details affected consumers’ behaviour around purchasing TA supplements. Future studies could be conducted to investigate the role of commercial information on websites selling HM with consumers’ HM-consuming behaviour.

The low-risk score received by the websites is reassuring. However, safety concerns towards the websites are still present due to the limited information concerning the potential risks of TA especially among high-risk consumers (e.g., chronic disease patients, elderly, pregnant women, and breastfeeding mothers). It should be noted that the risk assessment and the modified DISCERN tools used in this study had different criteria in assessing the aspects related to the risks of websites. Conclusions of the safety of websites that are based solely on one tool can be misleading. Therefore, it can be recommended to use both tools in a complementary manner in evaluating the risks of websites on HM. The present study showed that most health claims for TA supplements relate to the enhancement of testosterone and libido in men. Studies have shown that TA contains several biologically active substances such as eurycomanones and eurycomanols [4]. These substances may inhibit the conversion of testosterone to estradiol by the aromatase enzyme, thereby increasing testosterone levels and raising libido [47]. A recent review on the evidence of several DS, including TA as a testosterone booster, showed that at present there are insufficient data on their efficacy and safety [47]. Additionally, almost 35% of the websites claimed that TA supplements are beneficial for treating malaria. Although TA has been traditionally used against malaria [4], there is a lack of evidence of its efficacy as an antimalarial agent. Most studies relating to the effects of TA for the treatment of malaria are confined to animal or in vitro studies [48,49,50]. In summary, although TA may have potential clinical benefits around improving the levels of testosterone in men and other medical conditions, further efficacy trials should be conducted in order to make any firm recommendations for its use [15].

### Strengths and Limitations of the Study

One of the strengths of the study is the use of a modified DISCERN tool and risk score that allowed the assessment of the quality and risks of the websites. The findings from this study may provide insights into the level of quality and risks of websites about TA supplements, which is a commonly used HM among the public. Our findings should enable government agencies and HCPs to be more proactive in their critique and dissemination of information relating to HM. Moreover, efforts to raise Internet users’ awareness of how to identify good quality websites are warranted.

There are several limitations to this study. Firstly, in this study, an Internet search using the Google search engine was undertaken. The influence of temporal changes may limit the replicability of the study. Additionally, search results may vary based on the search terms used, time of which the search was conducted and country of origin settings within the search engine. It should be noted that the Internet search was conducted in March 2020. Since then, new websites on TA supplements may have been published, and the older ones may have been updated or removed, thus further minimizing the study’s replicability. Additionally, we excluded websites with complete access restricted by password, and those with personal opinions or experiences from social media, blogs, and Internet forums. Arguably, those sources may also contain meaningful information about TA supplements. However, in order to standardize evaluation and to allow comparability, those sources were excluded. Furthermore, we only included the first 50 websites for each search term combination used in the study. Therefore, relevant websites beyond the first 50 may have been missed. Nevertheless, based on the presumption relating to the predominant behavior of common Internet users, such a strategy may allow the identification of websites that are more likely to be visited by consumers [51].

Additionally, while the DISCERN tool is a validated tool to assess written information on treatment choices for a health problem, the version used in the present study is not validated. Despite this, a pilot test using the modified DISCERN tool prior to the actual study demonstrated its practicality. Moreover, in the present study, question 2 (*Does it achieve its aims?*) from the original DISCERN tool was removed. This was based on the performance of this item during the pilot test which revealed low usefulness in the study context. Of note, the original DISCERN tool stipulated that question 2 should be skipped if question 1 was provided with a rating of “1”. In this study, question 1 (*Are the aims clear?*) recorded a low mean score ± SD (1.14 ± 0.60), indicating that question 2 was irrelevant for the majority of websites. With that being said, future studies may retain question 2 if it is relevant to their study context and aims.

## 5. Conclusions

Websites containing information about TA supplements generally have a low-quality rating based on a modified DISCERN tool. However, the majority of these websites had a low-risk score with most of them not discouraging the use of conventional medicines or adherence to clinicians’ advice. Most health claims for TA supplements related to the enhancement of testosterone and libido, as well as treatment for malaria. Despite potential clinical benefits around improving the levels of testosterone in men, and in other conditions (e.g., malaria), further efficacy trials should be conducted in order to make any firm recommendation for its use. HCPs should be aware that TA supplement users may access information about TA from websites that could contain inadequate and unbalanced information about the supplements. Government agencies and HCPs must be more proactive in their critique and dissemination of information relating to HM, and in ensuring the safe use of HM among the public and patients.

## Figures and Tables

**Table 1:** Rating score for each of the modified DISCERN item.

Item Number	Modified DISCERN Item	Mean ± SD Score ^a^
1	Are the aims clear?	1.14 ± 0.60
2	Is it relevant?	2.29 ± 0.85
3	Is it clear what sources of information were used to compile the information on the website (other than the author or producer)?	2.31 ± 1.43
4	Is it clear when the information used or detailed on the website was produced?	2.26 ± 1.20
5	Is the information balanced and unbiased?	2.69 ± 1.24
6	Does the website provide details of additional sources of support and information?	1.15 ± 0.58
7	Does the website refer to areas of uncertainty?	1.05 ± 0.23
8	Does the website describe how TA works?	2.79 ± 1.31
9	Does the website describe the benefits of TA?	2.90 ± 1.26
10	Are drug-supplement interactions mentioned?	1.17 ± 0.63
11	Are contraindications of TA mentioned?	1.22 ± 0.65
12	Are potential adverse effects of TA mentioned?	1.53 ± 1.00
13	Is it clear from the website that there may be more than one possible treatment choice?	1.02 ± 0.16
14	Based on the answers to all of the above questions, rate the overall quality of the publication as a source of information about TA.	1.07 ± 0.51 ^b^

SD, standard deviation; TA, Tongkat Ali. ^a^ Based on a score of 1 to 5 (a score of 1 indicates a definite “no” i.e., the quality criterion has not been fulfilled at all; a rating from 2 to 4 indicates that the website partially meets the criterion in question, with a higher score indicating fewer shortcomings; and a score of 5 indicates a definite “yes”, i.e., the quality criterion has been completely fulfilled). ^b^ Based on a score of 1 to 5 (a rating from 1 to 2 indicates that the website has serious or extensive shortcomings; a rating of 3 to 4 indicates that the website has potentially important but not serious shortcomings; a score of 5 indicates that the website has minimal shortcomings).

**Table 2:** Health claims for TA in the websites.

Claims of Benefits	*n* (%)
Enhance testosterone level	121 (37.7)
Antimalaria	112 (34.9)
Improve libido	108 (33.6)
Treat infertility	89 (27.7)
Reduce stress	80 (24.9)
Enhance energy	59 (18.4)
Anticancer	57 (17.8)
Increase muscle gain	56 (17.4)
Treat fever	48 (15)

## Data Availability

The data presented in this study are available on request from the corresponding author.
